# Exploring the Desirable Attributes and Competencies of Pharmacy Clinical Preceptors: A Scoping Review

**DOI:** 10.3390/pharmacy13010005

**Published:** 2025-01-15

**Authors:** Haneen Alrawashdeh, Ahsan Sethi, Ahmed Awaisu, Banan Mukhalalati

**Affiliations:** 1 QU Health, Qatar University, Doha P.O. Box 2713, Qatar; ha091647@qu.edu.qa (H.A.); asethi@qu.edu.qa (A.S.); 2 Hamad Medical Corporation, Doha P.O. Box 3050, Qatar; 3 College of Pharmacy, QU Health, Qatar University, Doha P.O. Box 2713, Qatar; aawaisu@qu.edu.qa

**Keywords:** pharmacy education, experiential learning, clinical education, preceptorship, attributes, questionnaires

## Abstract

Background: Experiential learning is a vital component of health-professional education. It provides students with the opportunity to apply their knowledge in real-life settings before becoming licensed practitioners. Preceptors (i.e., practice educators) play a crucial role in developing students’ professional skills and competencies, as well as shaping their attitude during their clinical training. Ensuring preceptors’ quality and preparedness is a key aspect in students’ experiential learning due to the important impact of the provided training on the quality of the students’ learning experience. There is a knowledge gap about the desired attributes of pharmacy preceptors in the Gulf region, specifically Qatar, highlighting the need to explore preceptors’ views on this topic. Purpose: The aim of this scoping review is to identify the available tools in the literature to explore the desirable attributes of pharmacy preceptors as clinical educators. The objectives are to explore the reported desirable attributes of clinical preceptors in the published literature and select and utilize an appropriate tool to identify the desirable attributes of pharmacy preceptors in Qatar. Methods: The scoping review was designed to identify the relevant original research articles, which were published in English language, utilizing CINAHL, ERIC, ProQuest, and PubMed databases. Key concepts were preceptorship, attributes, pharmacy, and tools. Quantitative and mixed-methods study designs were included. The included articles were summarized according to their design, setting, population, and outcomes. The validity of the used instruments in these studies was reported. Results: A total of six articles qualified for inclusion into the full screening and were used to inform the results of this review. Skills like being a role model, assessment, and feedback expertise were of the highly important attributes to different populations (i.e., students, graduates, and preceptors). The review revealed the need for more validated tools in pharmacy research to increase the knowledge about the desired qualities of preceptors. Finally, a list of the most reported attributes in the literature was created after grouping all the reported attributes into five categories: (1) knowledge, teaching, and presentation skills; (2) professionalism and development skills; (3) communication skills; (4) supportive mentoring; and (5) enthusiasm and interest. Conclusions: the top three identified attributes were related to communication, assessment and feedback, and professionalism. The results of this review demonstrated a lack of well-designed and validated tools in pharmacy research that can be used to explore the desirable attributes of pharmacy preceptors. This necessitates further research to develop and validate a new appropriate tool to ultimately understand the perceptions of pharmacy preceptors on this topic. Including more databases in the research could have enriched the findings.

## 1. Introduction

Experiential learning is an important component of most health-professional education programs. Experiential learning occurs when a responsible learner cognitively, affectively, and behaviorally processes knowledge, skills, and attitudes in a learning situation characterized by a high level of active involvement [1]. Experiential learning provides students with the opportunity to apply their knowledge in real life during clinical practice before becoming licensed practitioners. The responsibility of transferring medical knowledge into the minds of learners falls upon the shoulders of medical educators [2]. Preceptors (i.e., practice educators) play a crucial role in developing students’ professional practice skills and competencies, as well as shaping their attitude during their clinical training rotations [3,4,5]. Their role extends beyond traditional teaching and theoretical concepts; they serve as mentors, guides, and role models to their students and frame their personal and professional growth [2].

The quality of the training provided by the preceptors significantly impacts the students’ experiential training experience in pharmacy education. In addition, preceptors’ desirable attributes as mentors, educators, and role models have a vital role in producing high-quality future practitioners [6]. Preceptors are therefore regarded as an essential asset to the experiential education of all pharmacy students. Hence, ensuring the quality and preparedness of the preceptors is considered a key aspect in pharmacy students’ experiential learning and requires continuous support by the educational and practice institutions and other stakeholders [7]. The training of preceptors is required and encouraged by pharmacy organizations, and studies have demonstrated benefits from preceptor training and development programs [8]. The American Association of Colleges of Pharmacy (AACP) has identified preceptor development as a significant element of quality experiential education, and the need for this training has been identified [8].

Experiential education could be improved by ensuring that preceptors are interested in teaching and have the time to teach [8]. This improvement is achievable by recognizing and addressing the problems faced by the preceptors [9]. Several studies have investigated the roles, skills, and attributes of preceptors from both the preceptors’ and the students’ perspectives [4,5,8]. The findings of those studies were variable, but there was a consensus among those studies on the essential skills that preceptors should possess [8,10,11]. Linking theory to practice, professionalism, effective communication skills, and providing effective feedback were repeatedly identified in many studies as important characteristics for the preceptors to possess, regardless of the participants (i.e., students vs. preceptors), the practice setting, and the countries where these studies were conducted [9,11,12].

The published literature pertaining to preceptorship in various health-profession education programs showed that preceptors were not always aware of their exact roles and responsibilities, their institutional goals, and may not be formally trained as preceptors [13,14,15]. However, more research and focus on the desired attributes of pharmacy preceptors are needed globally, and specifically in the Gulf region. In light of these findings, this review was designed to explore the appropriate tools used in the literature to identify either the desirable attribute or attributes and the competencies of pharmacy preceptors, and to determine the reported desirable attributes and competencies of clinical preceptors in the published literature.

Attributes is a term used to describe the personal qualities of preceptors that help them in creating a positive environment for learning and achieving learning outcomes. These include traits like empathy, patience, accessibility, etc. [16]. Competencies, on the other hand, refers to specific skills, abilities, and the measurable knowledge needed to perform the role of preceptor successfully [17]. The specific objectives of the review are to explore the tools used in the literature to investigate the desirable attribute or attributes and the competencies of pharmacy preceptors (i.e., practice educators). Moreover, we intend to use literature findings to generate a list of the identified attributes and/or competencies of pharmacy preceptors.

## 2. Materials and Methods

### 2.1. Protocol and Registration

A scoping review was conducted to identify the available tools used in pharmacy research to explore the perspectives of pharmacy preceptors on the desirable attributes of pharmacy preceptors as practice educators. The registration number of this scoping review on Research Registry is (reviewregistry1740) [18]. This scoping review was written according to the PRISMA statement for scoping reviews (PRISMA-ScR) [19]. Research questions were formulated following the PCC (Population, Concept, Context) framework. The population was the pharmacy preceptors, the concept was the preceptors’ perceptions on the desired attributes and competencies of a pharmacy preceptor, and the context was pharmacy education.

### 2.2. Eligibility Criteria

The focus of this review was to identify articles that investigated the desirable attributes and/or competencies of pharmacy preceptors (i.e., practice educators) and to use literature findings to create a list of the identified attributes of pharmacy preceptors. The eligibility criteria included English primary research studies that investigated the desirable attributes or the attributes and competencies of preceptors as practice educators in pharmacy education. In addition, the studies had to be available online as full-text studies, and published during the last 20 years (i.e., 2003–2023). The initial research was carried out with no research limits, to ensure a comprehensive outcome. Most of the studies were found to be irrelevant after the initial screening. Therefore, the authors decided to restrict the timeframe to 20 years to reflect the expansion in experiential learning research. Article types such as reviews, opinion articles, essays, and conference papers were excluded. Reference lists of the included articles were searched for relevant papers to ensure a comprehensive literature review. Theses and dissertations were also excluded, because they risked being less scientifically rigorous due to a lack of peer review and remaining unpublished in subscription-based journals.

### 2.3. Information Sources

An extensive search strategy was developed to identify relevant literature from different databases, including CINHAL, ERIC, PubMed, and ProQuest. The Boolean connector (AND) was used to connect four key concepts, namely preceptorship, attributes, pharmacy, and tools. The keywords used in the preceptorship concept search were “pharmacy preceptors”, “mentor”, “clinical supervisor”, “clinical teacher”, “clinical educator”, “clerkship”, “specialist”, “teacher”, “tutor”, “instructor”. These were combined using (OR). The attributes concept was searched using the following keywords: “characteristics”, “role model”, “qualities”, “features”, “traits”, “properties”, “example”, “idol”. The keywords for this concept were combined by (OR). The keywords used in the pharmacy concept included “hospital pharmacist” OR “community pharmacist” OR “clinical pharmacist” OR “pharmac*” and were combined by (OR). The keywords used to search for the tools concept were “questionnaire”, “instruments”, “survey”, “form”, “assessment”, “evaluation”. The keywords for this concept were combined using (OR).

### 2.4. Selection of Sources of Evidence

Covidence software (https://www.covidence.org/, accessed on 7 January 2025) was used to manage the literature findings. A manual check for duplicates was carried out first, then followed by the software checking and deletion process. After that, two investigators worked on a title and abstract screening of the identified articles from the above-mentioned search strategy. Irrelevant articles were excluded. After that, two investigators independently assessed the eligibility of the studies and finished the full-text screening. The remaining research team worked on more rounds of full-text reviews to ensure that relevant studies with appropriate objectives were included in the review. Disagreements were resolved by consensus through meetings and discussions.

### 2.5. Data Charting Process and Data Items

An extraction sheet was designed to tabulate data from the included articles using a Microsoft Excel^®^ spreadsheet. The extracted data included: (1) article title, (2) authors, (3) year of publication, (4) journal of publication, (5) study design, (6) study setting, (7) population, (8) primary objectives, (9) study outcomes, (10) used tools information, (11) tool validity, reliability, and (12) availability. Data extraction sheet piloting was completed by two investigators on three sample articles from the included articles in this review. Complete data extraction was carried out by one investigator, after the successful piloting.

## 3. Results

**Figure 1:**
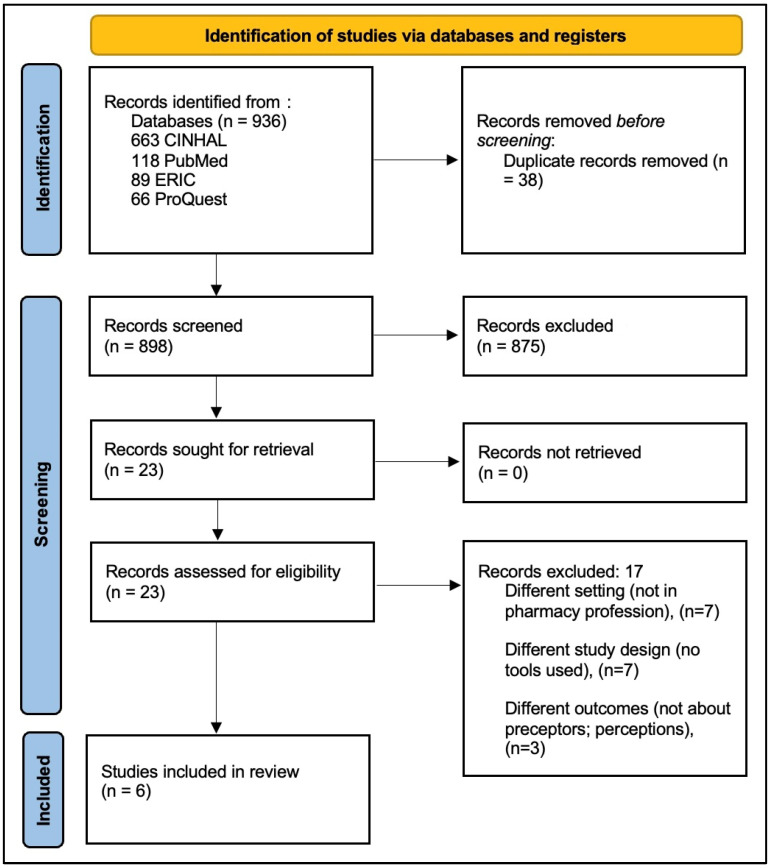
PRISMA flow diagram.

A narrative description of the included articles was summarized in tables to present the collected data. Out of 936 articles retrieved from databases, 38 were duplicates and were hence removed (Figure 1). After the title and abstract screening of 898 articles, 23 articles were eligible to be included in the full-text screening. The main reasons for exclusion were a different setting (i.e., the studies were not in pharmacy colleges or pharmacy training sites like hospitals and community pharmacies), a different study design which did not include a tool/survey for data collection (e.g., the Delphi method or qualitative designs that included interviews or focus groups), and different outcomes (i.e., studies that did not investigate perceptions and focused on other outcomes such as performance or satisfaction). A total of six articles qualified for inclusion into the full screening and were used to inform the results of this scoping review.

### 3.1. Characteristics of Included Studies

Of the six included studies, two studies were conducted in Australia, with one study in each of the following countries: Croatia, China, Sudan, and the USA. All the included studies used a cross-sectional design, except for one study, which used a mixed-methods design. The objectives of the included studies were to acquire insights into the ideal attributes of pharmacy preceptors from the various participant populations. The total number of participants in the included studies was 969, including a diverse array of levels in the professions (i.e., pharmacy mentors, pharmacy postgraduates, community pharmacists, pharmacy residents, university staff members including associate professors, assistant professors, professors, and lecturers). Table 1 demonstrates the characteristics of the included studies. In Table 2, the strengths and limitations of the included studies are summarized.

Both Alrakaf and Knott et al. were conducted in Australia and investigated the perceptions of students of their preferred teacher qualities, and the ideal roles and attributes of pharmacy preceptors [11,20]. The studies followed different designs; Alrakaf et al.’s study was conducted using survey of two measures: the achievement goal questionnaire (AGQ) and the build-a-teacher task [20]. Knott et al.’s study was designed as mixed-methods research, where the survey was followed by qualitative, semi-structured focus group interviews [11]. In Alrakaf et al.’s study there was consensus on the most and the least important traits. Both studies identified enthusiasm and good communication as important traits [20]. The quantitative and qualitative findings of Knott et al. revealed strong agreement on the ideal preceptor attributes, with good communication, enthusiasm for the profession, and the provision of clear and honest student feedback at the top of the most important attributes [11].

Three of the included articles were cross-sectional studies conducted to evaluate the perceptions of postgraduates, mentors, residents, and pharmacy teachers. The first study by Bochenek et al. evaluated pharmacy residents’ perception of preceptors as role models at the university of Kentucky [21]. There was no significant difference between the proportion of residents who thought it was important for a preceptor to be a role model and the proportion who perceived their current preceptors as a role model. Yue et al.’s study, which was designed to gain an idea about the development of pharmacy mentors’ competence by comparing differences between mentors’ and postgraduates’ perspectives at Chinese universities and colleges, concluded that good mentors should have three core competencies: professional knowledge, research competence, and communication competence [23]. In Sudan, the perception and commitment of pharmacy teachers in Sudanese governmental universities about their educational roles and responsibilities were assessed by Elseddig et al., using a tool that was adapted from the Association for Medical Education in Europe (AMEE) Guide No. 20, which states that a good teacher is more than a lecturer, encompassing twelve roles [24]. The guide looked at teaching and its components and explored the various responsibilities of educators in medical and health-profession education beyond teaching.

Half of pharmacy educators were found to have a good perception and good commitment regarding their role. In addition, a significant association was found between the perception and practice of the participants. In all the studies, the tools which were used were either piloted or validated tools.

Only one cross-sectional study had evaluated the perceptions of community pharmacists, which was conducted in Croatia. The self-assessed competencies of community pharmacist preceptors were evaluated and competencies for improvement were identified using the Croatian Competency Framework (CCF). Pharmacists assessed themselves as most competent in competencies related to “Organization and management”, while they assessed their competency in “Pharmaceutical public health competencies” as least competent. Table 3 presents the main findings and tool-related information of all the included studies.

### 3.2. List of the Identified Attributes

Assessing the outcomes of the included articles guided the development of a list of the attributes which were reported or ranked as important for the clinical preceptors to have. First, all the attributes from each paper were listed, then categorized in groups. Five categories were created to include the listed attributes: (1) knowledge, teaching, and presentation skills; (2) professionalism and development skills; (3) communication skills; (4) supportive mentoring; and (5) enthusiasm and interest. Under each category, attributes were gathered based on similarity. The complete list is shown in Table 4.

## 4. Discussion

### 4.1. Students’ Perceptions

This review has contributed valuable findings to the available literature on the desirable attributes of clinical pharmacy preceptors. Perceptions regarding the most important traits of a practice educator varied between different populations (i.e., students, postgraduates, residents, preceptors, etc.). According to the students’ viewpoints, linking theory to practice and focusing on students’ assessments in terms of the practical skills, such as communication and professionalism, were at the top of the desired traits [10]. This aligns with previous literature that identifies the preceptors’ need to maintain and improve their skills and expand their clinical knowledge, for the purpose of research and developing themselves as teachers or preceptors [25]. Interpersonal communication skills are crucial for the students to develop and practice during their training rotations in preparation for their role in practice. It is the responsibility of the preceptors to assist the students in identifying these skills and improving their performance during training before joining real practice settings. Preceptors are expected to guide the students in improving skills such as verbal and non-verbal communication, documentation, patient education and counseling, and appropriate communication with colleagues and other healthcare providers [26].

It was predicted that students would identify enthusiasm and expertise as crucial skills for a preceptor, while traits such as a reasonable workload and a warm personality will be considered as less important [20]. These findings align with the findings of the study conducted by Young, which reported showing interest in teaching (i.e., enthusiasm) and having a well-organized practice experience as the top factors that students have identified as strongly associated with excellent preceptors [8]. Nevertheless, students in Young et al.’s study did not perceive preceptor qualifications and knowledge as associated with preceptors’ excellence.

A closer look at the qualities of an excellent preceptor preferred by studies indicates that the overall highly valued qualities were those that reflected teacher engagement with the learning process [20]. This is consistent with the reported need in medical literature for preceptors to actively engage students. Treating students with trust and respect has also been identified as an effective teaching behavior [27]. Interestingly, the opposite was noted in a study from Ethiopia. Students gave a lower rating to tasks like encouraging the students to raise questions, discuss, and share opinions. Also, expressing care and being supportive to the students was given a lower rating by the students when compared to the preceptors [28].

### 4.2. Residents’ and Preceptors’ Perceptions

On the other hand, residents and preceptors rated being a role model, organization, and management-related competencies as the highest competencies for a clinical educator [18]. In Bochenek et al.’s study, which was conducted at the University of Kentucky to evaluate pharmacy residents’ perception of preceptors as role models, residents identified areas for preceptor improvement, including having a well-organized and structured rotation and providing good direction and feedback [21]. These characteristics have a high impact on the overall understanding of the residents about the expectations from the rotations. Also, good feedback supports the learning and growth of residents as practitioners. A lack of these characteristics in preceptors may affect their role as role models to their learners [21]. These findings are consistent with the findings of Hartzler et al.’s study, which was conducted in United States (U.S.) to assess the status of pharmacy preceptor development. According to Hartzler et al., many institutions lacked site policies, or preceptors were not always aware of them. The availability of clear instructions (i.e., policies) makes training requirements clearer and more applicable [25]. In addition, preceptors must be able to provide criteria-based feedback and an evaluation of resident performance [29]. Over 90% of the surveyed preceptors in Hartzler et al.’s study indicated feeling comfortable conducting performance evaluations and providing the residents with clear and effective feedback. This is also supported by the findings of an Ethiopian study, where the preceptors gave a higher rating, compared to the students, to acting as a role model to their students and management-related tasks.

### 4.3. Community Pharmacists’ Perceptions

Držaić et al. presented anticipated findings from community pharmacists. Community pharmacy preceptors perceived themselves as least competent in activities that involved direct patient care (i.e., patient-oriented) under the “Public health” and “Pharmaceutical care” clusters. This perception is expected, as community pharmacies have always been seen, in different parts of the world, as businesses which prioritize profit [22]. Furthermore, giving high scores for the “Dispensing medicines and medical devices” competency compared to lower scores for the “Pharmaceutical care competency” cluster, with the lowest scores in the “Assurance of safe medicines use” competency, stands in agreement with the traditional role of community pharmacists, which is known to be more drug oriented [22]. Similar findings were reported in Mills et al.’s study, where community pharmacists perceived themselves to be more competent in the “provision of drug product” competencies compared to other competencies [30]. The abovementioned perceptions of community pharmacists do not reflect the “teacher” competency in the World Health Organization (WHO)’s concept of the “Seven-Star Pharmacist”. A pharmacist has the responsibility to assist with the education and training of future generations of pharmacists [31].

### 4.4. Faculty Perceptions

Mentors and pharmacy faculty perception differs when evaluating their work or self-assessing their competency. Communicating knowledge and sharing information with the learners is a fundamental part of teaching [32]. The literature revealed that mentors and pharmacy educators were, most of the time, more concerned about competencies such as the ability to teach in classrooms and to act as role models to the learners [24]. In addition, they rated organization and management-related competencies as highly important competencies for mentors [23]. These perceptions vary depending on many factors like the adopted curriculum model and years of experience [24]. The faculties of institutions that educate following the teacher-centered curriculum are expected to concentrate more on competencies that help them to deliver the knowledge to their learners efficiently. On the other hand, faculties who work in schools that follow student-centered curriculum are expected to focus on competencies like communications skills, providing feedback appropriately, and the capability to integrate technology within the learning process [2].

### 4.5. Tools Availability

Although all the included studies have utilized different tools to achieve their aims, none of these tools was appropriate to explore preceptors’ viewpoints about the desirable attributes of pharmacy clinical preceptors. Most of the identified work in the field was more related to continuous professional development, preceptors’ training programs, and pharmacists’ self-assessment tools [4,22,25]. Future research and studies should consider the need for more reliable tools to identify preceptors’ perceptions in the field of pharmacy clinical education and experiential learning.

### 4.6. Other Healthcare Professions

Like pharmacy, other healthcare professions such as those that are medicine-, nursing-, or nutrition require well-prepared preceptors with important qualities needed to produce future professionals with the required level of knowledge, skills, and competencies [33]. Acting as a role model to the students and supporting them, professionalism, and enthusiasm for teaching were of the most recurring attributes of a preceptor within pharmacy [33]. The literature from a nursing viewpoint revealed that preceptors were expected to enhance students’ knowledge and skills, orient the students and help them engage in the learning environment, and assist them in acquiring and developing necessary skills such as time management, reflection, problem solving, and critical thinking [15]. Dietitians also reported the importance of skills related to planning, evaluating, teaching, and facilitation for a preceptor to play the role effectively [14]. Communication skills, teaching and evaluation skills, and building a relationship with the students were constantly necessary competencies in health-profession education, regardless of the profession.

This review provided useful findings about the perceptions of pharmacy preceptors regarding the desirable attributes of pharmacy preceptors as practice educators and reviewed the published tools that were used in the literature to achieve this aim. However, some limitations should be highlighted when interpreting the findings of this review. For example, this review was limited to four databases and to the time before the year 2000, which results in potentially missing relevant articles in other major databases (ex. Scopus and Cochrane) and those published before 2000. In addition, qualitative studies were excluded, although such studies may provide deeper views and opinions. Despite these limitations, the review findings summarized the available information on the research topic, which added to the available, related literature.

## 5. Conclusions

This review was designed to answer these two questions: “What are the appropriate tools used in the literature to identify the desirable attributes of pharmacy preceptors?” and “What are the reported desirable attributes of clinical preceptors in published literature?”. No appropriate published tools were identified in pharmacy-related articles which evaluated the pharmacy preceptors’ attributes and characteristics. Most of the papers were related to development and improvement, as well as preceptors’ training programs. Thus, this review adds to the literature and highlights the need for future research in the field of pharmacy focusing on the perceptions of pharmacy preceptors regarding the necessary traits of practice educators, especially in Qatar and the surrounding region. Moreover, a list of the reported attributes of pharmacy preceptors was developed based on the review findings. In conclusion, more validated tools need to be utilized in future pharmacy studies in the field of experiential learning and clinical education.

## Figures and Tables

**Table 1:** The characteristics of the included studies.

Author	Year	Participants	Setting	Country	Study Design	Main Objectives
Alrakaf [20]	2014	366 2nd- and 4th- year pharmacy students	The University of Sydney’s Faculty of Pharmacy	Australia	A two-measures survey: the Achievement Goal Questionnaire (AGQ) and the build-a-teacher task	To investigate the relationships between pharmacy students’ preferred teacher qualities and their academic achievement goal orientations
Bochenek [21]	2016	69 pharmacy residents	University of Kentucky	USA	Cross-sectional	To evaluate pharmacy residents’ perception of preceptors as role models
Držaić [22]	2018	223 community pharmacists	Community pharmacies in Croatia	Croatia	Cross-sectional	To evaluate the self-assessed competencies of community pharmacist preceptors, and to identify competencies to be improved
Yue [23]	2020	236 pharmacy mentors and postgraduates	Chinese universities and colleges	China	Cross-sectional	To acquire insight into the development of pharmacy mentors’ competence by comparing differences between mentors’ and postgraduates’ perspectives
Elseddig [24]	2022	125 staff members (i.e., lecturers, assistant professors, associate professors, and professors)	Governmental pharmacy colleges in Sudan	Sudan	Cross-sectional	To assess the perception and commitment of pharmacy teachers about their roles and educational responsibilities
Knott [11]	2022	68 students in levels 2, 3, and 4 of the BPharm (Hons) program	Australian University and James Cook University (JCU)	Australia	Mixed-methods study; survey followed by focus group interviews	To investigate student perceptions of the preceptors’ ideal roles and attributes to inform the development of a preceptor training program

**Table 2:** The strengths and limitations of the included studies.

Author	Year	Strengths	Limitations
Alrakaf [20]	2014	Using two validated measuring instruments Using an engaging method to determine students’ preferences for teacher qualities	Using a cohort from one institution may affect the generalizability of the findings
Bochenek [21]	2016	The adapted tool was pilot tested Areas of improvement were identified	The demographics of preceptors were provided by residents (i.e., the study participants) The timing of survey distribution Using a cohort from one institution may affect the generalizability of the findings Participants evaluated only one preceptor, even if they had multiple preceptors
Držaić [22]	2018	Using a validated framework High response rate (85.8%) The findings may guide the improvement of mentor training programs	The participants of the study are not representative of the general pharmacy population Using self-assessment as an outcome measure, which may be influenced by many factors and lead to unreliable ratings
Yue [23]	2020	High response rate (95%) Using a well-structured instrument with good reliability, ensuring the consistency of the results	The study was limited to the Chinese system of pharmacy education
Elseddig [24]	2022	Using an adapted questionnaire from the validated AMEE guide No. 20 Diversity of participants	Including a limited number of universities Factors affecting the perceptions of pharmacy teachers were not assessed
Knott [11]	2022	Using a mixed-methods approach provided breadth and depth of information Representative sample	Students from one university may not be representative of the wider population of pharmacy students Potential response bias (i.e., the principal researcher was involved in student teaching)

**Table 3:** The key findings of the included studies.

Author	Year	Key Findings	Data Collection Tool
Alrakaf [20]	2014	Consensus on the most important traits (i.e., enthusiasm, and clear presentation style) and the least important traits (i.e., interactive teaching, and reasonable workload)	The AGQ is a validated and psychometrically robust instrument Validated
Bochenek [21]	2016	A total of 86% of residents viewed their preceptors as role models No significant difference between the proportion of residents who perceived their preceptor as a role model and the proportion who thought it was important for preceptor to be role model	A 55-item survey instrument was developed, pilot tested, and distributed to pharmacy residents in Kentucky Piloted
Držaić [21]	2018	Most competent in competencies pertaining to the cluster “Organization and management competencies” Least competent in the competencies pertaining to the cluster “Pharmaceutical public health competencies”	The Croatian Competency Framework (CCF) Validated
Yue [23]	2020	Good mentors should possess three core competencies: research competence, professional knowledge, and communication competence	The ‘Research on the competence of pharmacy professional mentors in Chinese universities’ questionnaire Validated
Elseddig [24]	2022	The most important role perceived was a lecturer in the classroom setting A significant association was found between the perception and practice of the participants	An online self-administered questionnaire adapted from the Association for Medical Education in Europe (AMEE) Guide No. 20: a good teacher is more than a lecturer, encompassing twelve roles Validated
Knott [11]	2022	Key role of preceptors: linking theory to practice and to focus regarding students’ assessments on practical skills such as communication and professionalism Strong agreement between the quantitative and qualitative findings on the ideal preceptor attributes Good communication, enthusiasm for the profession, and the provision of clear and honest student feedback thought to be the most important attributes	The survey questions were informed by the aim of the study and a review of the literature to identify potential preceptor roles and recommended skills and attributes Piloted

**Table 4:** The themes of the identified attributes of preceptors in the included studies.

Author	Year	Themes			
		Teaching and Presentation Skills	Knowledge and Development Skills	Communication Skills	Enthusiasm and Support
Alrakaf [20]	2014	√	√		√
Bochenek [21]	2016	√	√		√
Držaić [22]	2018		√		√
Yue [23]	2020		√	√	√
Elseddig [24]	2022	√	√		
Knott [11]	2022	√	√	√	√

## Data Availability

The deidentified raw data supporting the conclusions of this article can be made available by the authors on request.
